# Cycling on the Freeway: The perilous state of open-source neuroscience software

**DOI:** 10.1162/imag_a_00554

**Published:** 2025-05-02

**Authors:** Britta U. Westner, Daniel R. McCloy, Eric Larson, Alexandre Gramfort, Daniel S. Katz, Arfon M. Smith, Arnaud Delorme, Vladimir Litvak, Scott Makeig, Robert Oostenveld, Jan-Matthijs Schoffelen, Tim M. Tierney

**Affiliations:** Department of Medical Neuroscience, Donders Institute for Brain, Cognition and Behaviour, Radboud University Medical Center, Nijmegen, The Netherlands; Donders Centre for Cognition, Donders Institute for Brain, Cognition and Behaviour, Radboud University, Nijmegen, The Netherlands; Institute for Learning and Brain Sciences, University of Washington, Seattle, WA, United States; Université Paris-Saclay, Inria, CEA, Palaiseau, France; NCSA & Siebel School of Computing and Data Science & iSchool, University of Illinois Urbana-Champaign, Champaign, IL, United States; GitHub Inc., San Francisco, CA, United States; Swartz Center for Computational Neuroscience (SCCN), Institute of Neural Computation (INC), University of California, San Diego, CA, United States; Centre de Recherche Cerveau et Cognition (CerCo), Centre National de la Recherche Scientifique (CNRS), Paul Sabatier University, Toulouse, France; Department of Imaging Neuroscience, UCL Queen Square Institute of Neurology, London, United Kingdom; Donders Centre for Cognitive Neuroimaging, Donders Institute for Brain, Cognition and Behaviour, Radboud University, Nijmegen, The Netherlands; NatMEG, Karolinska Institutet, Stockholm, Sweden

**Keywords:** open science, open source, neuroscience, electrophysiology

## Abstract

Most scientists need software to perform their research (Barker et al., 2020;Carver et al., 2022;Hettrick, 2014;Hettrick et al., 2014;Switters & Osimo, 2019), and neuroscientists are no exception. Whether we work with reaction times, electrophysiological signals, or magnetic resonance imaging data, we rely on software to acquire, analyze, and statistically evaluate the raw data we obtain—or to generate such data if we work with simulations. In recent years, there has been a shift toward relying on free, open-source scientific software (FOSSS) for neuroscience data analysis (Poldrack et al., 2019), in line with the broader open science movement in academia (McKiernan et al., 2016) and wider industry trends (Eghbal, 2016). Importantly, FOSSS is typically developed by working scientists (not professional software developers), which sets up a precarious situation given the nature of the typical academic workplace wherein academics, especially in their early careers, are on short- and fixed-term contracts. In this paper, we argue that the existing ecosystem of neuroscientific open-source software is brittle, and discuss why and how the neuroscience community needs to come together to ensure a healthy software ecosystem to the benefit of all.

## 1 The Neuroscience Open Source System is Moving toward a Crisis

The development and maintenance of open-source scientific software is labor-intensive, especially when adopting best practices of open science: readability, resilience, and re-use (Connolly et al., 2023). Here (and throughout) when we say “Open-source software” or “FOSSS,” we are referring to larger software packages, not the openly shared project code of single researchers or research teams. While the latter is an important and useful contribution to Open Science, the former comes with unique challenges regarding project administration and management, software maintenance (often across different operating systems), and catering to a large user base. Critically, the survival of any open-source software is guaranteed not only by the total number of contributors, but also by the number of developers who have a sufficiently complete overview of the source code and the tools and processes in place for maintenance of the project. The*Truck Factor*(or*Bus Factor*) quantifies the number of such maintainers for a given project, and can be seen as an indicator of the risk of incomplete knowledge- and capability-sharing among the project’s team members (Avelino et al., 2016).Box 1shows the Truck Factors of several widely used analysis software projects in electrophysiological neuroscience. It is evident that projects typically have a Truck Factor of one to three, meaning that only one to three people per project have sufficient knowledge to keep the project alive (note that the Truck Factor does not take into account whether those contributors with the relevant knowledge are still active or have funding/support to do the maintenance work). This reveals the fragility of the academic open-source system:*all users*of a software package rely on the work of*one to three people*to keep everyone’s data analyses from failing. It also highlights the enormous pressure the maintainers of open-source software are under. Given the importance of scientific software to the practice of modern science, one would hope developing FOSSS would be a well-supported and incentivized role in academia. This hope is not borne out: the academic incentive structure for software development and maintenance is woefully inadequate (Carver et al., 2022;Davenport et al., 2020;Merow et al., 2023;Smith et al., 2018) and can even be perceived as hostile (Millman & Pérez, 2018;Pérez, 2011). AsDavenport et al. (2020)state: “In spite of the vital role research software plays, it largely remains undervalued, with time spent in training or development seen as detracting from the ‘real research’.” As maintainers and developers of neuroscience FOSSS (see caption ofBox 1for details) and researchers of the open-source movement in academia, we strongly agree with this notion: The current incentive model often summarized as “publish-or-perish” has a significant negative impact on the software ecosystem’s health in several ways, which we will describe below.

**Table:** 

**Software**	**Truck factor**	**Commit hash**
Brainstorm	1	3ffdc9687e7ca8386ce2ddcc072dfa1cac8a5e9
EEGLAB	1	87f3cffd91912cce6e2015baeb7f2d11f8953134
FieldTrip	1	b01eeefcf9a10a6726d91237459c8607a3c344cb
MNE-Python	3	96679b70ce34b970f5225131e2b609ad59a599f
SPM	3	a169eb951b45a2162c06a5febdb4004980eab356
Astropy	6	62018574e74cda9c4b30b088f4049e01fb403fbd

**Brainstorm**(Tadel et al., 2011) is a comprehensive application dedicated to multimodal brain data analysis and visualization (MEG, EEG, fNIRS, intracranial and invivo/latex invitro electrophysiology, MRI, and CT). It features both a rich, intuitive graphical user interface, and a powerful library of process functions that can be combined into pipeline scripts. As of April 2024, Brainstorm registered*>*43,000 user accounts and was featured in*>*3,400 research articles. In the one-year period ending 24 June 2024, it had 213 commits from 13 contributors.

**EEGLAB**(Delorme & Makeig, 2004) is an interactive MATLAB toolbox for the analysis of EEG, MEG, and other electrophysiological data. It provides a graphical user interface as well as a structured programming environment. It is developed at the Swartz Center for Computational Neuroscience and has been cited more than 10,000 times over 20 years (Fayaz, 2024). In the one-year period ending 24 June 2024, it had 226 commits from 6 contributors.

**FieldTrip**(Oostenveld et al., 2011) is a MATLAB software toolbox for MEG, EEG, and iEEG analysis, and was initiated in 2004 by Robert Oostenveld, soon thereafter joined by Jan-Mathijs Schoffelen, who remain the main contributors to date. The toolbox is used by thousands of researchers. In the one-year period ending 24 June 2024, it had 424 commits from 10 contributors.

**MNE-Python**(Gramfort et al., 2013) was started in 2010 by Alexandre Gramfort as a Python port of Matti Hämäläinen’s MNE software (written in C). Since then, its community has grown to include more than 350 contributors and thousands of users. In the one-year period ending 24 June 2024, it had 564 commits from 28 contributors.

Statistical Parametric Mapping (**SPM**) is open-source software for analyzing PET, MRI, and MEG/EEG/OPM data. It has been in continuous development for over 30 years, since its introduction in the early 1990s by Karl Friston. SPM for M/EEG (Litvak et al., 2011) is noted for its elaborate set of tools for parametric statistical testing as well as for Bayesian source analyses and biophysically informed dynamic causal modeling (DCM). Development has recently transitioned to GitHub, coordinated by the Methods Group at the Functional Imaging Laboratory (FIL) in London. In the one-year period ending 24 June 2024, it had 215 commits from 14 contributors.

**Astropy**is a core package for astronomy and astrophysics research. The project started in 2011 in an effort to provide a unified Python package for the different subfields of astronomy (The Astropy Collaboration et al., 2013). In the one-year period ending 24 June 2024, it had 1749 commits from 33 contributors.

**Box 1:** The reported Truck Factors (top) were estimated using the heuristic-based approach reported inAvelino et al. (2016). This algorithm estimates the Truck Factor using a degree-of-authorship metric (Fritz et al., 2010): “authors” of files are those developers who are able to maintain a file moving forward. Developers who have (joint) authorship of at least 50% of files count toward the Truck factor. For each package, the Truck Factor analysis was performed 25 June 2024; the commit hash for the state of the codebase when we performed the analysis is shown. We compare neuroscience packages to Astropy (The Astropy Collaboration et al., 2013), a well-supported and widely used software package in astronomy and astrophysics.

Note that the Truck Factor only quantifies maintainer contributions with respect to changes in the codebase and does not reflect other important open-source contributions such as community management, bug reports and triaging, project management, etc. Assessing Truck Factors accurately is difficult and we note that some of these might be underestimated, while others seem to be overestimated. However, an estimate between 1 and 3 seems realistic for each of the neuroscience FOSSS projects we report on here.

Software descriptions (bottom) feature commit and contributor counts of the one-year period ending 24 June 2024. Commits were counted using the git log function, and the number of contributors was identified using the GitHub contributors interface.

Note that several of the authors of this paper are maintainers or developers of the reported projects: Authors Westner, McCloy, Larson, and Gramfort maintain MNE-Python. Authors Delorme and Makeig maintain EEGLAB. Authors Litvak and Tierney maintain SPM. Authors Oostenveld and Schoffelen maintain FieldTrip.

## 2 Rewarding Publications in Promotion Disfavors Software Development

Many scientists who develop software publish a paper to introduce their project to the community, and direct users to cite that paper if they use the software for their analysis (Katz & Chue Hong, 2018;Smith et al., 2016). However, software papers are usually published to mark the first public release or sometimes a later major release of a software package. Given that publication counts are such an important (and often the primary) metric for academic tenure and promotion, once the paper announcing the software is published, there is little incentive from the employer to continue developing the software. This may be changing as academic institutions rely more on composite citation metrics rather than simple publication counts—by incentivizing that the paper be widely cited—but the reward of new projects and attendant new publications can easily outweigh the reward of garnering more software-paper citations, especially given that software papers are viewed as less valuable than research papers (Davenport et al., 2020). At the same time, people who contribute to development or maintenance of FOSSS at a later stage do not get rewarded by citations of the initial paper (Davenport et al., 2020), thus increasing the chance that the software will become unmaintained. We worry that the lack of benefit for project “latecomers” also incentivizes starting new projects instead of contributing to existing ones—since a new project means a new publication—which, in turn, risks further increasing the number of unmaintained software packages in the field. To spell it out: open-source work done by academics (in the time they might otherwise use to do research) sustains*other*academics writing*their*papers (Merow et al., 2023)—which puts the software-developing academics behind in the publish-or-perish culture of academia.

## 3 Lack of Stable Long-Term Funding for Scientific Software Stifles its Growth

Funding is hard to obtain for the development of new software—and even harder for the maintenance of existing software (Davenport et al., 2020). For example, currently in the US National Institutes of Health (NIH) funding system, proposals to continue development of open software are reviewed alongside new-software proposals and are ranked on “Innovation”, which leads to uncompetitive scores for maintenance projects. That means that many contributors and maintainers either do not have secure long-term academic positions or are not paid primarily for their software work. This is slowly changing: more and more programs^1^are supporting the development of FOSSS. However, such grants are often promoting novelty, that is, the addition of new features or the launch of new software projects, not the maintenance of existing projects. Once the grant is over, the primary incentive to continue maintaining the software is largely gone, a void quickly filled by the ever-present publish-or-perish incentive lurking in the background.

## 4 Existing Barriers Skew the Developer Demographic to the Detriment of Science

Research shows that diversity has positive effects on project outcomes generally (Earley & Mosakowski, 2000;Hoogendoorn et al., 2013;Jackson & Joshi, 2004;Roberson, 2019) and for open-source projects in particular (Daniel et al., 2013;Vasilescu, Posnett, et al., 2015). However, structural factors such as misogyny and racism hinder diversity of the developer pool: only 1.1–5.4% of open-source developers are perceptible as or identify as women (Eghbal, 2016;Geiger, 2022;Ghosh et al., 2002;Nafus, 2012) and less than 17% are perceptible as Non-White (Nadri et al., 2021). The cost of contributing is higher for minorities (Whitaker & Guest, 2020): not only do they often feel a pressure to have to be perfect (Singh & Bongiovanni, 2021), but also members of underrepresented groups have to face stereotyping, discrimination, and harassment in the open-source software world (Frluckaj et al., 2022;Nadri et al., 2021;Nafus, 2012;Singh & Bongiovanni, 2021;Vasilescu, Filkov, & Serebrenik, 2015).

We are unaware of any systematic studies examining diversity in neuroscience software, but our collective experience in the field suggests that it is not substantially different from the broader open-source software community. If true, this is unsurprising: academics need a certain level of privilege to be able to contribute to open source in the first place. Typical barriers to participation in FOSSS projects are the lack of permission and support from a supervisor, the lack of “free” time outside normal work hours due to family or financial demands on that time, and lack of confidence due to inadequate training, role models, and guidance. These barriers all affect marginalized groups more strongly, and are compounded by the lack of representation and role models from underrepresented demographics and by the myth of meritocracy (Nafus, 2012). Moreover, the “informalization” of open source (Nafus, 2012) compounds the problem by eschewing traditional application and advancement processes and legal workplace protections in the spaces and interactions where FOSSS work is carried out, and consequently such spaces are often dominated by “old boys” networks where again underrepresented identities face an uphill battle. Diversity in age and career stage of FOSSS contributors and maintainers is also an issue. Many academic open-source contributors are pursuing or have just finished their Ph.D. and the devaluing of software work makes it harder for them to achieve tenured positions, while more senior academics who contributed early in their career are often not able to prioritize open-source work anymore, due to the publish-or-perish culture and lack of stable funding for software development in academia.

Each of these barriers to achieving a diverse community of FOSSS contributors and maintainers exacerbates the substantial lack of qualified labor (cf.Box 1).

In summary, workplace incentives in academia disfavor participation in open-source communities; the lack of a level playing field further discourages participation for certain classes of academics; and changing the incentives through extramural funding has so far been a temporary, partial fix on too small a scale to be transformative. But given the shift toward relying on FOSSS in neuroscience, it seems clear that the incentive structure must continue to evolve. In the following, we will discuss ways in which such change can be realized.

## 5 Professionalize Academic Software Development

One obvious cornerstone for a more sustainable and professional FOSSS ecosystem is funding, especially funding that supports maintenance of existing, widely-used projects (Merow et al., 2023). That funding should support*scientists*who engage in software development as part of their normal academic appointment duties. It has further been suggested that all levels of software development in academia (from widely-used packages down to single-user analysis scripts) can be carried out or supported by experienced research software engineers (Chapuis & Winter, 2024;Connolly et al., 2023;Merow et al., 2023). As others have noted, there are at least two obstacles to academic software work being carried out by professional software engineers: first, academia struggles to attract technically skilled professionals given the (much) higher compensation available in industry (Connolly et al., 2023;Gewin, 2022;Seidl et al., 2016), and second, there is a perception among professional engineers that career advancement prospects in academia are limited (Carver et al., 2022;Connolly et al., 2023;Merow et al., 2023). However, even if institutional support for research software engineering were to increase dramatically, we see a further obstacle to the sustainability of FOSSS: academic software development needs the participation of scientists that are active in the field and have the domain knowledge necessary to understand relevant use cases, best practices, and common analysis pitfalls. This latter point also underscores the importance of having more senior academics involved in software development, which (as discussed above) is disincentivized and, consequently, rare. Thus, we believe that a robust solution must involve institutions increasing their support of*working scientists*who develop software (perhaps alongside professional research software engineers), and recognizing and rewarding software contributions as an integral part of the practice of modern science.

## 6 Adopting new Software Citation Practices can Increase the Valuation of FOSSS

Increasing the valuation of open-source work for academic career advancement is crucial. One previously suggested, yet debatable, shortcut to this would be the exploitation of the existing publish-or-perish culture by introducing “update publications” after software development milestones (Merow et al., 2023). However, in our view this risks perpetually re-creating the same set of problems, just on a shorter time-scale. Encouraging researchers to cite the software they use—and to cite the*software itself*(i.e., the specific version used), not the canonical paper that introduced the software—seems like a better adaptation within the existing culture: it does not require the developers to write a new paper, and all contributors to a specific version of the software get credit. Indeed, “cite the software, not the paper” is considered best practice already (Katz et al., 2020;Smith et al., 2016), and we hope that this can become standard practice in neuroscience; developers can make this easy by assigning persistent identifiers like DOIs to each release using services like Zenodo, but publishers, journals, editors, and reviewers must play a role here in insisting that authors attribute their software usage in line with those best practices.

## 7 Start Viewing Research Software Work as an Important Contribution to Science

We urge funding bodies and promotion committees to value substantial open-source software work in their evaluation guidelines as an important contribution to science of equal value as one or (ideally) multiple papers. The development of widely used analysis software should be acknowledged and rewarded as a contribution to science rather than viewed as merely the development of one’s coding skills. Individual investigators can play an important role here too: if your lab relies on FOSSS for data analysis, consider allocating grant funds to support a scientific software developer, making FOSSS contribution part of the job description for your next postdoc or research scientist hire, or planning that your graduate students will need either a longer duration of support or fewer research output expectations (or both) in order to develop the necessary competencies*to both use and contribute to*the tools they rely on.

Importantly, it should not only be the developer’s responsibility to prove the value of their contributions; the burden should lie with promotion and tenure committees to become familiar with how to evaluate the scope and import of software contributions (fortunately, the Research Software Alliance and Research Data Alliance are working on policy recommendations on this topic,^2^and CURIOSS is working to support and improve open-source program offices across academia^3^). Failing that, promotion and tenure committees should, at minimum, allow, request, and encourage scientists who develop software to contextualize the scope and impact of their software work (Hafer & Kirkpatrick, 2009). We, however, want to caution against the development of new and too simplistic metrics for the measure of open-source work in academia, as measuring software impact is difficult and takes time (Afiaz et al., 2023). Furthermore, Goodhart’s law postulates that every measure that becomes a target becomes a bad measure (Afiaz et al., 2023;Goodhart, 1984), as has happened to the*h-index*(e.g.,Bartneck & Kokkelmans, 2011;Purvis, 2006;Seeber et al., 2019;Zhivotovsky & Krutovsky, 2008).

## 8 Better Training can Facilitate a Healthy FOSSS Ecosystem in the Long-Term

Lastly, we advocate for programs and departments to incorporate better training of students and PhD candidates in software development and maintenance (Carver et al., 2022;Guest & Forbes, 2023;Millman & Pérez, 2018): this would not only increase the software engineering skills of academics generally (Connolly et al., 2023), but would also send a strong signal about the importance of a well-written, reusable code to junior researchers. While training programs such as, for example, Software Carpentry^4^, CodeRefinery^5^, INTERSECT^6^, Neurohackademy^7^, URSSI^8^, or Neuromatch Academy^9^, give excellent training courses and summer schools, we also call on teaching coordinators of universities to reflect an increasing need for programming literacy in their neuroscience programs. Together with the recognition for writing open-source software, this would be an important step toward a healthy software ecosystem in neuroscience.

## 9 A Higher Valuation of FOSSS will Increase the Inclusivity of Open-Source Spaces

We predict that all action points discussed above will have a positive impact on the inclusivity of open-source work in academia. Changing the incentive structure such that open-source work does not have to be a privilege anymore but gets seen as what it is, a valid and critical contribution to science, will facilitate the participation of underrepresented groups. Beyond this, we strongly advocate that open-source projects in neuroscience make sure to be welcoming to everyone and to prevent any harassment, for example, by stating and adhering to Community Guidelines.

## 10 Conclusion

Summarizing the key points of this paper, we hope to raise awareness within the neuroscientific community about its dependence on a rather brittle structure. Your open-source software ecosystem needs your help! Immediate action can be taken by citing current software versions instead of the seminal software-describing paper, by making space for your trainees to engage in FOSSS communities, and by rewarding them (or at least not penalizing them) when they do. Beyond this, the incentive structure in academia and the policies that support it (including those created by research-performing organizations, funders, publishers, etc.) urgently need to be re-thought (Hostler, 2023;Jensen & Katz, 2023;Merow et al., 2023;Millman & Pérez, 2018;Munafò et al., 2017;Neylon et al., 2012)—not only for the sake of the academic open-source ecosystem, but for the good of the neuroscience community as a whole.

## Data Availability

This paper does not rely on any custom code. All resources used for the Truck Factor analysis are reported inBox 1.

## Footnotes

1 e.g., US National Science Foundation’s POSE program and CSSI “Transition to Sustainability” programs, stand-alone software- and developer/maintainer-focused grants as well as open science supplements to research grants from the US National Institutes of Health, the UK Research and Innovation office, the national plan for open science (“plan national pour la science ouverte”) of the French government, and software-focused grants from organizations like CZI, the Sloan Foundation, and the Simons Foundation

2
https://www.rd-alliance.org/groups/policies-research-organisations-research-software-pro4rs

3
https://curioss.org/

4
https://software-carpentry.org/

5
https://coderefinery.org/

6
https://intersect-training.github.io

7
https://neurohackademy.org

8
https://urssi.us/projects/urssi-software-school/

9
https://academy.neuromatch.io

## References

[b1] Afiaz , A. , Ivanov , A. A. , Chamberlin , J. , Hanauer , D. , Savonen , C. L. , Morgan , M. , Reich , M. , Getka , A. , Holmes , A. , Pati , S. , Boutros , P. C. , Bakas , S. , Caporaso , J. G. , Fiol , G. D. , Haas , B. , Schloss , P. D. , Eddy , J. A. , Albrecht , J. , Fedorov , A. , … Wright , C. ( 2023 ). Evaluation of software impact designed for biomedical research: Are we measuring what’s meaningful? arXiv . https://arxiv.org/abs/2306.03255

[b2] Avelino , G. , Passos , L. , Hora , A. , & Valente , M. T. ( 2016 ). A novel approach for estimating truck factors . 2016 IEEE 24th International Conference on Program Comprehension (ICPC) , 1 – 10 . 10.1109/ICPC.2016.7503718

[b3] Barker , M. , Katz , D. S. , & Gonzalez-Beltran , A. ( 2020 ). Evidence for the importance of research software . Zenodo . 10.59350/mnnj6-at315

[b4] Bartneck , C. , & Kokkelmans , S. ( 2011 ). Detecting h-index manipulation through self-citation analysis . Scientometrics , 87 ( 1 ), 85 – 98 . 10.1007/s11192-010-0306-5 21472020 PMC3043246

[b5] Carver , J. C. , Weber , N. , Ram , K. , Gesing , S. , & Katz , D. S. ( 2022 ). A survey of the state of the practice for research software in the United States . PeerJ Computer Science , 8 , e963 . 10.7717/peerj-cs.963 PMC9138129 35634111

[b6] Chapuis , G. , & Winter , O. ( 2024 ). Neuroscience needs a career path for software engineers . 10.53053/xgrg8885

[b7] Connolly , A. , Hellerstein , J. , Alterman , N. , Beck , D. , Fatland , R. , Lazowska , E. , Mandava , V. , & Stone , S. ( 2023 ). Software engineering practices in academia: Promoting the 3Rs—Readability, Resilience, and Reuse . Harvard Data Science Review , 5 ( 2 ). 10.1162/99608f92.018bf012

[b8] Daniel , S. , Agarwal , R. , & Stewart , K. J. ( 2013 ). The effects of diversity in global, distributed collectives: A study of open source project success . Information Systems Research , 24 ( 2 ), 312 – 333 . 10.1287/isre.1120.0435

[b9] Davenport , J. H. , Grant , J. , & Jones , C. M. ( 2020 ). Data without software are just numbers . Data Science Journal , 19 , 3 . 10.5334/dsj-2020-003

[b10] Delorme , A. , & Makeig , S. ( 2004 ). EEGLAB: An open source toolbox for analysis of single-trial EEG dynamics including independent component analysis . Journal of Neuroscience Methods , 134 ( 1 ), 9 – 21 . 10.1016/j.jneumeth.2003.10.009 15102499

[b11] Earley , C. P. , & Mosakowski , E. ( 2000 ). Creating hybrid team cultures: An empirical test of transnational team functioning . Academy of Management Journal , 43 ( 1 ), 26 – 49 . 10.5465/1556384

[b12] Eghbal , N. ( 2016 ). Roads and bridges: The unseen labor behind our digital infrastructure . https://www.fordfoundation.org/wp-content/uploads/2016/07/roads-and-bridges-the-unseen-labor-behind-our-digital-infrastructure.pdf

[b13] Fayaz , M. ( 2024 ). The bibliometric analysis of EEGLAB software in the Web of Science indexed articles . Neuroscience Informatics , 4 ( 1 ), 100154 . 10.1016/j.neuri.2023.100154

[b14] Fritz , T. , Ou , J. , Murphy , G. C. , & Murphy-Hill , E. ( 2010 ). A degree-of-knowledge model to capture source code familiarity . Proceedings of the 32nd ACM/IEEE International Conference on Software Engineering -Volume 1 , 385 – 394 . 10.1145/1806799.1806856

[b15] Frluckaj , H. , Dabbish , L. , Widder , D. G. , Qiu , H. S. , & Herbsleb , J. D. ( 2022 ). Gender and participation in open source software development . Proceedings of the ACM on Human-Computer Interaction , 6 ( CSCW2 ), 1 – 31 . 10.1145/3555190 37360538

[b16] Geiger , R. S. ( 2022 ). Summary analysis of the 2017 GitHub open source survey . 10.17605/OSF.IO/ENRQ5

[b17] Gewin , V. ( 2022 ). Has the ‘Great resignation’ hit academia? Nature , 606 , 211 – 213 . 10.1038/d41586-022-01512-6 35641675

[b18] Ghosh , R. A. , Glott , R. , Krieger , B. , & Robles , G. ( 2002 ). The free/libre and open source software survey and study—FLOSS final report. https://www.researchgate.net/publication/264799695_The_freelibre_and_open_source_software_survey_and_study-FLOSS_final_report

[b19] Goodhart , C. A. ( 1984 ). Problems of monetary management: The UK experience . Springer . 10.1007/978-1-349-17295-5_4

[b20] Gramfort , A. , Luessi , M. , Larson , E. , Engemann , D. A. , Strohmeier , D. , Brodbeck , C. , Goj , R. , Jas , M. , Brooks , T. , Parkkonen , L. , & Hämäläinen , M. ( 2013 ). MEG and EEG data analysis with MNE-Python . Frontiers in Neuroscience , 7 , 267 . 10.3389/fnins.2013.00267 24431986 PMC3872725

[b21] Guest , O. , & Forbes , S. H. ( 2023 ). Teaching coding inclusively: If this, then what? (Preprint). SocArXiv. 10.31235/osf.io/3r2ez

[b22] Hafer , L. , & Kirkpatrick , A. E. ( 2009 ). Assessing open source software as a scholarly contribution . Communications of the ACM , 52 ( 12 ), 126 – 129 . 10.1145/1610252.1610285

[b23] Hettrick , S. ( 2014 ). It’s impossible to conduct research without software, say 7 out of 10 UK researchers. Software and Research , 5 , 1536 . https://www.software.ac.uk/blog/its-impossible-conduct-research-without-software-say-7-out-10-uk-researchers

[b24] Hettrick , S. , Antonioletti , M. , Carr , L. , Hong , N. C. , Crouch , S. , Roure , D. C. D. , Emsley , I. , Goble , C. , Hay , A. , Inupakutika , D. , Jackson , M. , Nenadic , A. , Parkinson , T. , Parsons , M. I. , Pawlik , A. , Peru , G. , Proeme , A. , Robinson , J. , & Sufi , S. ( 2014 ). UK Research Software Survey 2014 . 10.1145/2618137.2618140

[b25] Hoogendoorn , S. , Oosterbeek , H. , & Van Praag , M . ( 2013 ). The impact of gender diversity on the performance of business teams: Evidence from a field experiment . Management Science , 59 ( 7 ), 1514 – 1528 . 10.1287/mnsc.1120.1674

[b26] Hostler , T. J. ( 2023 ). The invisible workload of open research . Journal of Trial and Error , 4 ( 1 ). 10.36850/mr5

[b27] Jackson , S. E. , & Joshi , A. ( 2004 ). Diversity in social context: A multi-attribute, multilevel analysis of team diversity and sales performance . Journal of Organizational Behavior , 25 ( 6 ), 675 – 702 . 10.1002/job.265

[b28] Jensen , E. A. , & Katz , D. S. ( 2023 ). Charting the course: Policy and planning for sustainable research software . https://urssi.us/blog/2023/06/22/introducing-charting-the-course-policy-and-planning-for-sustainable-research-software/

[b29] Katz , D. S. , & Chue Hong , N. P. ( 2018 ). Software citation in theory and practice . In J. H. Davenport , M. Kauers , G. Labahn , & J. Urban (Eds.), Mathematical software – ICMS 2018 (pp. 289 – 296 , Vol. 10931 ). Springer International Publishing . 10.1007/978-3-319-96418-8_34

[b30] Katz , D. S. , Hong , N. P. C. , Clark , T. , Muench , A. , Stall , S. , Bouquin , D. , Cannon , M. , Edmunds , S. , Faez , T. , Feeney , P. , Fenner , M. , Friedman , M. , Grenier , G. , Harrison , M. , Heber , J. , Leary , A. , MacCallum , C. , Murray , H. , Pastrana , E. , … Yeston , J. ( 2020 ). Recognizing the value of software: A software citation guide . F1000Research , 9 , 1257 . 10.12688/f1000research.26932.2 33500780 PMC7805487

[b31] Litvak , V. , Mattout , J. , Kiebel , S. , Phillips , C. , Henson , R. , Kilner , J. , Barnes , G. , Oostenveld , R. , Daunizeau , J. , Flandin , G. , Penny , W. , & Friston , K. ( 2011 ). EEG and MEG data analysis in SPM8 . Computational Intelligence and Neuroscience , 2011 , 1 – 32 . 10.1155/2011/852961 21437221 PMC3061292

[b32] McKiernan , E. C. , Bourne , P. E. , Brown , C. T. , Buck , S. , Kenall , A. , Lin , J. , McDougall , D. , Nosek , B. A. , Ram , K. , Soderberg , C. K. , Spies , J. R. , Thaney , K. , Updegrove , A. , Woo , K. H. , & Yarkoni , T. ( 2016 ). How open science helps researchers succeed . eLife , 5 , e16800 . 10.7554/eLife.16800 27387362 PMC4973366

[b33] Merow , C. , Boyle , B. , Enquist , B. J. , Feng , X. , Kass , J. M. , Maitner , B. S. , McGill , B. , Owens , H. , Park , D. S. , Paz , A. , Pinilla-Buitrago , G. E. , Urban , M. C. , Varela , S. , & Wilson , A. M. ( 2023 ). Better incentives are needed to reward academic software development . Nature Ecology & Evolution , 7 ( 5 ), 626 – 627 . 10.1038/s41559-023-02008-w 36849538

[b34] Millman , K. , & Pérez , F. ( 2018 ). Developing open-source scientific practice . In V. Stodden , F. Leisch , & R. D. Peng (Eds.), Implementing Reproducible Research ( 1st ed., pp. 149 – 183 ). Chapman and Hall/CRC . 10.1201/9781315373461-6

[b35] Munafò , M. R. , Nosek , B. A. , Bishop , D. V. M. , Button , K. S. , Chambers , C. D. , Percie Du Sert , N., Simonsohn , U. , Wagenmakers , E.-J. , Ware , J. J. , & Ioannidis , J. P. A. ( 2017 ). A manifesto for reproducible science . Nature Human Behaviour , 1 ( 1 ), 0021 . 10.1038/s41562-016-0021 PMC7610724 33954258

[b36] Nadri , R. , Rodriguezperez , G. , & Nagappan , M. ( 2021 ). On the relationship between the developer’s perceptible race and ethnicity and the evaluation of contributions in OSS . IEEE Transactions on Software Engineering , 1 – 1 . 10.48550/arXiv.2104.06143

[b37] Nafus , D. ( 2012 ). ‘Patches don’t have gender’: What is not open in open source software . New Media & Society , 14 ( 4 ), 669 – 683 . 10.1177/1461444811422887

[b38] Neylon , C. , Aerts , J. , Brown , C. T. , Coles , S. J. , Hatton , L. , Lemire , D. , Millman , K. J. , Murray-Rust , P. , Perez , F. , Saunders , N. , Shah , N. , Smith , A. , Varoquaux , G. , & Willighagen , E. ( 2012 ). Changing computational research. The challenges ahead . Source Code for Biology and Medicine , 7 ( 1 ), 2 . 10.1186/1751-0473-7-2 22640749 PMC3441321

[b39] Oostenveld , R. , Fries , P. , Maris , E. , & Schoffelen , J.-M. ( 2011 ). FieldTrip: Open source software for advanced analysis of MEG, EEG, and invasive electrophysiological data . Computational Intelligence and Neuroscience , 2011 , 1 – 9 . 10.1155/2011/156869 21253357 PMC3021840

[b40] Pérez , F. ( 2011 ). Ten years of (interactive) scientific Python .

[b41] Poldrack , R. A. , Gorgolewski , K. J. , & Varoquaux , G. ( 2019 ). Computational and informatic advances for reproducible data analysis in neuroimaging . Annual Review of Biomedical Data Science , 2 ( 1 ), 119 – 138 . 10.1146/annurev-biodatasci-072018-021237

[b42] Purvis , A. ( 2006 ). The h index: Playing the numbers game . Trends in Ecology & Evolution , 21 ( 8 ), 422 . 10.1016/j.tree.2006.05.014 16781007

[b43] Roberson , Q. M. ( 2019 ). Diversity in the workplace: A review, synthesis, and future research agenda . Annual Review of Organizational Psychology and Organizational Behavior , 6 ( 1 ), 69 – 88 . 10.1146/annurev-orgpsych-012218-015243

[b44] Seeber , M. , Cattaneo , M. , Meoli , M. , & Malighetti , P. ( 2019 ). Self-citations as strategic response to the use of metrics for career decisions . Research Policy , 48 ( 2 ), 478 – 491 . 10.1016/j.respol.2017.12.004

[b45] Seidl , A. , Wrzaczek , S. , El Ouardighi , F. , & Feichtinger , G. ( 2016 ). Optimal career strategies and brain drain in academia . Journal of Optimization Theory and Applications , 168 ( 1 ), 268 – 295 . 10.1007/s10957-015-0747-3

[b46] Singh , V. , & Bongiovanni , B. ( 2021 ). Motivated and capable but no space for error: Women’s experiences of contributing to open source software . The International Journal of Information, Diversity, & Inclusion (IJIDI) , 5 ( 3 ). 10.33137/ijidi.v5i3.36197

[b47] Smith , A. M. , Katz , D. S. , Niemeyer , K. E. , & FORCE11 Software Citation Working Group . ( 2016 ). Software citation principles . PeerJ Computer Science , 2 , e86 . 10.7717/peerj-cs.86

[b48] Smith , A. M. , Niemeyer , K. E. , Katz , D. S. , Barba , L. A. , Githinji , G. , Gymrek , M. , Huff , K. D. , Madan , C. R. , Cabunoc Mayes , A. , Moerman , K. M. , Prins , P. , Ram , K. , Rokem , A. , Teal , T. K. , Valls Guimera , R. , & Vanderplas , J. T. ( 2018 ). Journal of Open Source Software (JOSS): Design and first-year review . PeerJ Computer Science , 4 , e147 . 10.7717/peerj-cs.147 PMC7340488 32704456

[b49] Switters , J. , & Osimo , D. ( 2019 ). Recognising the importance of software in research: Research Software Engineers (RSEs), a UK example. https://op.europa.eu/en/publication-detail/-/publication/fd0f6775-e0dd-11e9-9c4e-01aa75ed71a1

[b50] Tadel , F. , Baillet , S. , Mosher , J. C. , Pantazis , D. , & Leahy , R. M. ( 2011 ). Brainstorm: A user-friendly application for MEG/EEG analysis . Computational Intelligence and Neuroscience , 2011 , 1 – 13 . 10.1155/2011/879716 21584256 PMC3090754

[b51] The Astropy Collaboration , Robitaille , T. P. , Tollerud , E. J. , Greenfield , P. , Droettboom , M. , Bray , E. , Aldcroft , T. , Davis , M. , Ginsburg , A. , Price-Whelan , A. M. , Kerzendorf , W. E. , Conley , A. , Crighton , N. , Barbary , K. , Muna , D. , Ferguson , H. , Grollier , F. , Parikh , M. M. , Nair , P. H. , … Streicher , O. ( 2013 ). Astropy: A community Python package for astronomy . Astronomy & Astrophysics , 558 , A33 . 10.1051/0004-6361/201322068

[b52] Vasilescu , B. , Filkov , V. , & Serebrenik , A. ( 2015 ). Perceptions of diversity on Git Hub: A user survey . 2015 IEEE/ACM 8th International Workshop on Cooperative and Human Aspects of Software Engineering , 50 – 56 . 10.1109/CHASE.2015.14

[b53] Vasilescu , B. , Posnett , D. , Ray , B. , van den Brand , M. G. , Serebrenik , A. , Devanbu , P. , & Filkov , V. ( 2015 ). Gender and tenure diversity in GitHub teams . Proceedings of the 33rd Annual ACM Conference on Human Factors in Computing Systems , 3789 – 3798 . 10.1145/2702123.2702549

[b54] Whitaker , K. , & Guest , O. ( 2020 ). #Bropenscience is broken science . https://www.bps.org.uk/psychologist/bropenscience-broken-science

[b55] Zhivotovsky , L. A. , & Krutovsky , K. V. ( 2008 ). Self-citation can inflate h-index . Scientometrics , 77 ( 2 ), 373 – 375 . 10.1007/s11192-006-1716-2

